# Protection of Cochlear Ribbon Synapses and Prevention of Hidden Hearing Loss

**DOI:** 10.1155/2020/8815990

**Published:** 2020-11-01

**Authors:** Mei Wei, Wei Wang, Yao Liu, Xiang Mao, Tai Sheng Chen, Peng Lin

**Affiliations:** ^1^Department of Otorhinolaryngology Head and Neck Surgery, Tianjin First Central Hospital, 300192 Tianjin, China; ^2^Institute of Otolaryngology of Tianjin, China; ^3^Key Laboratory of Auditory Speech and Balance Medicine, Tianjin, China; ^4^Key Clinical Discipline of Tianjin (Otolaryngology), China; ^5^Otolaryngology Clinical Quality Control Centre, Tianjin, China

## Abstract

In the auditory system, ribbon synapses are vesicle-associated structures located between inner hair cells (IHCs) and spiral ganglion neurons that are implicated in the modulation of trafficking and fusion of synaptic vesicles at the presynaptic terminals. Synapse loss may result in hearing loss and difficulties with understanding speech in a noisy environment. This phenomenon happens without permanent hearing loss; that is, the cochlear synaptopathy is “hidden.” Recent studies have reported that synapse loss might be critical in the pathogenesis of hidden hearing loss. A better understanding of the molecular mechanisms of the formation, structure, regeneration, and protection of ribbon synapses will assist in the design of potential therapeutic strategies. In this review, we describe and summarize the following aspects of ribbon synapses: (1) functional and structural features, (2) potential mechanisms of damage, (3) therapeutic research on protecting the synapses, and (4) the role of synaptic regeneration in auditory neuropathy and the current options for synapse rehabilitation.

## 1. Introduction

Hair cells (HCs) in the mammalian cochlea play a vital role in converting mechanical sound waves into neural signals for hearing [1–3]. Ribbon synapses are the vital structures between inner HCs (IHCs) and spiral ganglion neurons (SGNs) and are the first excitatory afferent synapses in the auditory pathway. Acoustic stimuli of various intensities and frequencies cause corresponding changes in the number, structure, shape, and function of ribbon synapses. These synapses are highly sensitive to noise, ototoxic drugs, inflammation, and aging [4–9], factors that can lead to changes in the hearing threshold.

In the traditional sense, “hearing loss” (i.e., an increase in threshold) is generally considered to result from damaged HCs [10–15]. However, in recent years, clinical and basic studies have shown that an increase in the threshold value does not necessarily accompany hearing loss; after a period of time, the threshold value returns to the normal level [16–19]. A special type of sensorineural hearing loss, namely, “hidden hearing loss” (HHL), can be caused by damaged synapses between IHCs and SGNs; no dysfunction in the auditory threshold is observed, yet serious perceptual difficulties are obvious, especially in understanding speech in a noisy environment [20, 21]. HHL reportedly correlates with the loss of a subset of synaptic connections between the IHCs and the auditory nerve (AN) [22, 23]. To better elucidate the effect of synaptic damage on hearing and to provide new perspectives and clues to explain clinically complex hearing problems, in this article, we summarize the mechanisms underlying ribbon synaptic damage, protection, and regeneration.

## 2. Functional and Structural Features of Ribbon Synapses

Ribbon synapses play an important role in the inner ear system. They are vesicle-associated structures implicated in the modulation of trafficking and fusion of synaptic vesicles at the presynaptic terminals [24]. In the mammalian auditory system, ribbon synapses are essential for phase locking and spatial sound localization [25]. In a vestibular HC, ribbon synapses play a role in triggering the ms-range vestibulo-ocular reflex [26]. In the inner ear system, the shape, size, and number of ribbon synapses vary according to the spatial position and physiological state, even at different developmental stages of the same animal [27]. Ribbon synapses of vestibular HCs have a smaller number of ribbons and a tighter coupling with Ca^2+^ channels compared with cochlear IHCs [28]. In the IHCs, the ribbon synapse structure differs in the high- and low-frequency regions of the cochlea, leading to variations in the exocytosis of ribbon synapses in terms of spatial distribution and calcium dependence [27].

**Figure 1:**
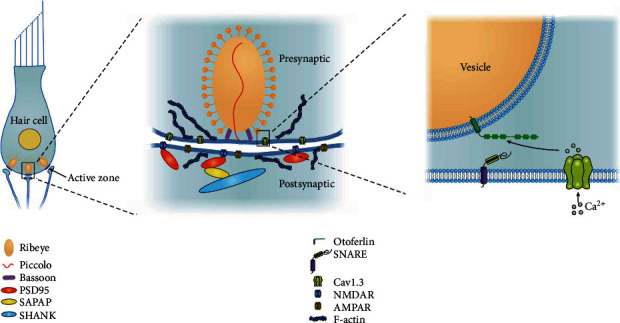
Schematic of the cochlear IHC synapse.

Recently, the structure and function of ribbon synapses have been further studied. Proteins play essential roles in mammalian IHC synapses, including Ribeye, Otoferlin, Cav1.3 channels, Bassoon, Piccolo, SHANK, PSD95, and F-actin (Figure 1).

Ribeye is the main protein forming the framework of the ribbon, and it can regulate ribbon size. Ribeye is a splice variant of the transcriptional corepressor CtBP2 and has two domains, the unique A domain and the B domain. The B domain includes a nicotinamide (NAD^+^, NADH, or NAD(H))-binding domain. In developing HCs, NAD^+^ promotes Ribeye-Ribeye interactions or Ribeye localization to the ribbon, whereas NADH inhibits them. Ribbon size is directly regulated by the levels of NAD^+^ and NADH [29].

Otoferlin is a unique calcium sensor that is involved in calcium-regulated synaptic transmission as well as vesicle release in vestibular and cochlear synapses [30]. Otoferlin is a multivalent protein capable of simultaneously binding multiple copies of the cytoplasmic loop of Cav1.3 and SNARE (soluble N-ethylmaleimide-sensitive factor attachment protein receptor complex) proteins. Otoferlin acts as a calcium-sensitive scaffolding protein, localizing SNARE proteins proximal to the calcium channel. This close apposition permits fast membrane fusion and exocytosis of neurotransmitters in response to sound [31].

Cav1.3 channels are the main L-type Ca^2+^ channel subunits involved in synaptic exocytosis in IHCs [32]. The domains II and III of Cav1.3, consisting of amino acids 752–891, are referred to as loop 1.3 [33]. The loop 1.3 region of voltage-gated calcium channels is thought to contribute to exo- and endocytotic processes through interaction with multiple Otoferlin C2 domains [34]. The membrane-associated Cav1.3 channels aggregate in small clusters in close spatial organization to the synaptic ribbons, forming a spatially restricted Ca^2+^ microdomain in the active zone [35].

Bassoon and Piccolo are two significant scaffolding proteins of the assembled cytomatrix. They play important roles in the maintenance of the presynaptic structure and function and in the assembly of synaptic ribbons. These two proteins can regulate activity-dependent communication between presynaptic boutons and the neuronal nucleus [36], as well as specific protein ubiquitination and proteasome-mediated proteolysis, which potentially induces short-term plasticity at the presynapse. Bassoon and Piccolo also induce molecular rearrangements at the presynapse, with the reprogramming of the expression of neuronal activity-regulated genes. These two large scaffolding proteins use their modular structure to organize supermolecular complexes essential for various aspects of presynaptic function [36].

Receptor clusters can be found in the postsynaptic terminal of the ribbon synapses, including *α*-amino-3-hydroxy-5-methyl-4-isoxazolepropionic acid (AMPA) receptors (AMPARs) and N-methyl-D-aspartate (NMDA) receptors (NR1, NR2A/B). Mechanosensory HCs release glutamate at the ribbon synapses to excite postsynaptic afferent neurons via AMPA-type ionotropic glutamate receptors (AMPARs) [37]. NMDA receptors are present at the afferent synapses in the cochlea, which can modulate the reaction of AMPAR to glutamate at the type I afferent terminals [38].

The SHANK protein family, including SHANK1, SHANK2, and SHANK3, comprises “master” scaffolding proteins in the postsynaptic site. The proteins interconnect many components of the postsynaptic density with those of the cytoskeletal matrix, including NMDA, AMPA, and metabotropic glutamate receptors (mGluRs) [39]. AMPA transmission may be affected by the deletion of SHANK genes. SHANK proteins play a pivotal role in synaptic development and function and also in the regulation of excitatory neurotransmission [40].

PSD95, an important regulator of synaptic maturation, is an essential scaffolding protein in synaptogenesis and neurodevelopment. It interacts with, stabilizes, and directs N-methyl-D-aspartic acid receptors (NMDARs) and AMPARs to the postsynaptic membrane [41]. PSD95 is perhaps the best characterized MAGUK protein of the postsynaptic density. It has a PDZ domain that can interact with NMDARs, which potentially affects glutamate transmission and the formation of silent synapses during critical time points of neurodevelopment. The C-terminus of the protein has a guanylate kinase domain, which is linked to members of the SAPAP family of proteins. SAPAP proteins subsequently bind to the SHANK protein family. The protein of PSD95 can serve as an interface between clustered membrane-bound receptors, cell adhesion molecules, and the actin cytoskeleton [42].

In auditory HCs, the pressure produced by sound waves is converted into an electrical nerve signal. Ribbon synapses are essential in neural encoding of microphonic potentials by mechanisms involving Cav1.3 channels and Otoferlin-dependent exocytosis of synaptic vesicles [28, 43]. The F-actin network maintains tight spatial organization of Cav1.3 Ca^2+^ channels and influences the efficiency of Otoferlin-dependent exocytosis. It controls the flow of synaptic vesicles during exocytosis at the IHC ribbons, thus playing a vital role in the function of normal ribbon synapses [44].

The unique morphological, molecular, and physiological characteristics of ribbon synapses help to maintain high rates of neurotransmitter release, with a high degree of temporal precision. At each synapse, an electron-dense ribbon is located in the presynaptic region. The ribbon is normally surrounded by a halo of synaptic vesicles containing glutamate [45]. The presynaptic membrane is depolarized after external sound stimulation, resulting in the generation of a depolarizing receptor potential of the potassium cation influx. This graded potential triggers Ca^2+^ influx through voltage-gated Ca^2+^ channels (VGCCs) at the presynaptic active zones of the ribbon synapse. Calcium in the presynaptic active zones binds to the HC's Ca^2+^ sensor and Otoferlin, driving synaptic vesicle fusion and glutamate release [46]. Glutamate released into the synaptic cleft then activates AMPA receptors present on the terminals of the auditory afferent nerve fibers, thereby completing the excitatory transmission of sound signals on the AN.

## 3. Mechanisms of Ribbon Synapse Damage

Studies have shown that the number of cellular synapses can be influenced by acute noise exposure, ototoxicity, gene mutations, and aging. However, the number of IHCs does not change. An increasing number of detailed studies have been conducted to determine the causes and molecular-level mechanisms of cochlear synaptic lesions.

### 3.1. Genetic Factors

Recently, genetic studies have examined the molecules required for hearing in zebrafish and mammals. Of the synapse-associated genes, *DFNB9* and *Cav1*.*3*, in particular, are required for HC neurotransmission. Mutations in these genes have been associated with HHL. Here, we discuss the influence of these mutants on HC synaptic development, maintenance, and function.

#### 3.1.1. *DFNB9*

**Figure 2:**
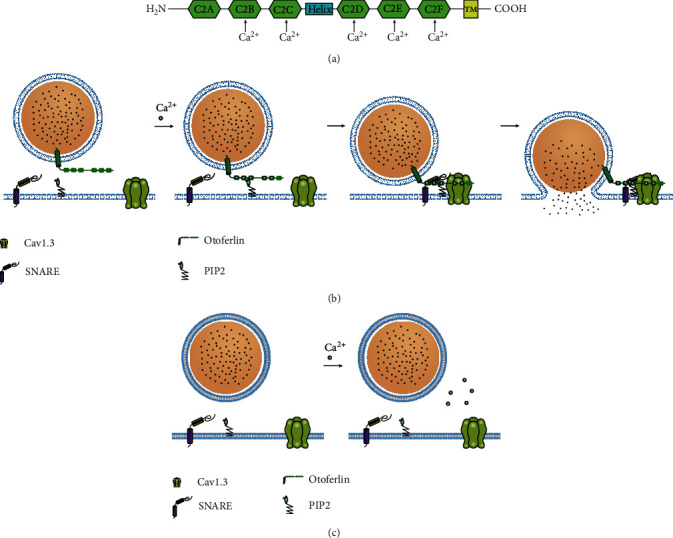
Schematic of the structure and function of Otoferlin. (a) Diagram of Otoferlin depicting the six C2 domains, labeled C2A–C2F, and the transmembrane domain (TMD). The binding of Ca^2+^ is indicated for all C2 domains with the exception of C2A. (b) The function of Otoferlin in vesicle fusion. (i) Ca^2+^-dependent phospholipid binding targets the presynaptic membrane via interaction with PIP2. (ii) Ca^2+^-dependent approximation of vesicular and plasma membranes. (iii) Interaction with proteins such as SNARE and the Cav1.3 channels to mediate Ca^2+^-triggered fusion. (c) Loss of Otoferlin. The extracellular transport of synaptic vesicles in the IHCs ceases completely.

The OTOF gene (*DFNB9*) encodes Otoferlin, a transmembrane protein belonging to the ferlin protein family that is expressed in cochlear IHCs and is needed for synaptic exocytosis at the ribbon synapse [47]. Otoferlin comprises multiple C2 domains (A–F) [48]. A C2 domain contains 100–130 amino acids and can mediate interaction with membranes by binding Ca^2+^ and negatively charged lipids such as phosphatidylserine (PS) or phosphatidylinositol 4,5-bisphosphate (PIP2). In a recent study of Otoferlin C2 domains, the binding of Ca^2+^ was indicated for all C2 domains with the exception of C2A [49, 50] (Figure 2(a)). The C2C and C2F domains of Otoferlin preferentially bind PIP2, and PIP2 may target Otoferlin to the presynapse in a calcium-independent manner [51]. This positioning facilitates fast calcium-dependent exocytosis at the HC synapse [48]. Otoferlin can trigger ultrafast exocytosis and endocytosis and can recruit synaptic vesicles to the active zone [49]. It acts as a scaffolding protein that first targets the presynaptic membrane by interacting with PIP2 and subsequently interacts with the calcium channels and membrane fusion machinery (one or more SNARE isoforms). The linking of the synaptic vesicle, presynaptic calcium channel, and membrane fusion proteins in close spatial proximity was shown to reduce the “reaction pace” and increase the fidelity and precision of exocytosis in response to presynaptic calcium influx [34]. The potential molecular mechanisms of Otoferlin function include (i) targeting the presynaptic membrane via interaction with PIP2, (ii) Ca^2+^-dependent approximation of vesicular and plasma membranes, and (iii) interaction with proteins such as SNARE and the Cav1.3 channels to mediate Ca^2+^-triggered fusion [52] (Figure 2(b)).

Pathogenic mutations in *OTOF* cause nonsyndromic autosomal recessive deafness DFNB9 [53]. To date, more than 160 variants of *OTOF* have been reported [54], and gene mutations are among the major causes of auditory neuropathy [55, 56]. *OTOF* mutations disrupt the functioning of the ribbon synapses by impairing multivesicular glutamate release. In Otof^−/−^ mice, the auditory brainstem response (ABR) test demonstrates that the amplitude of the *I* wave is reduced, and the band-shaped synaptic morphology of Otof^−/−^ mice is normal. However, the extracellular transport of synaptic vesicles in the IHCs ceases completely [47] (Figure 2(c)). Otoferlin operates as a Ca^2+^ sensor at the IHC synapse [57]. In Otoferlin knockout mice, the rapid phase of exocytosis is abolished in IHCs and is not reversed by fast Ca^2+^ influx or Ca^2+^ uncaging [58]. Mutation in C2C may induce decreased levels of Otoferlin, and exocytosis may be critically reduced during prolonged stimulation. Mutation in the C2F domain results in a slower replenishment of the readily releasable pool of vesicles at the synaptic pole of IHCs [33]. The C2D domain of Otoferlin interacts with the calcium channel Cav1.3 in a calcium-dependent manner. Mutation in C2D leads to substantially decreased binding to Cav1.3, independent of calcium [35]. For fast and precise acoustic coding, a large, rapidly releasable pool (RRP) of synaptic vesicles is crucial for the release of large amounts of neurotransmitters within a short time. Otoferlin could promote the formation of long tethers that hold and prime the synaptic vesicles in the IHC active zones, which are crucial structures for vesicle replenishment [48].

#### 3.1.2. *Cav1.3*

Cav1.3 channels belong to the L-type calcium channel family and play a role in neurotransmission at HC synapses. The Cav1.3 channels are ideal calcium channels for mediating rapid and continuous exocytosis, as they can be activated by lower voltages than those needed to activate other Cav1 channels [32]. Cav1.3 channels cluster tightly at synaptic ribbons and are important for tightly coupled calcium influx and vesicle release [59].

Cav1.3 Ca^2+^ channels have protein isoforms resulting from extensive alternative splicing, especially in the C-terminus region, thereby underlying their different gating properties [60]. The long and short C-terminal isoforms of the Cav1.3 Ca^2+^ channels differ in the kinetics of their Ca^2+^ channels and their relative sensitivity to the L-type Ca^2+^ channel blocker nifedipine. The short C-terminal isoforms encode the phasic exocytotic component and have low sensitivity to nifedipine; they control the fast fusion of the RRP. The expression of short fast inactivating isoforms of the Cav1.3 channels decreases in IHCs lacking Otoferlin [61]. The long isoforms of the Cav1.3 Ca^2+^ channel, with slow inactivation and great sensitivity to nifedipine, regulate the sustained or tonic exocytosis of the RRP. Slow inactivating long Cav1.3 isoforms enable sustained and deeper Ca^2+^ diffusion in IHCs, which is essential for the recruitment of vesicles located at large distances from the release sites [62]. In IHCs, the mechanisms of transient and sustained exocytosis use the different isoforms of the Cav1.3 channels [61]. The Cav1.3 L-type channels control IHC sensory function. Downregulated expression of Cav1.3 is related to age-related hearing loss. Cav1.3 protects cells in the auditory pathway from oxidative stress, so decreased Cav1.3 expression can lead to hearing loss through the enhancement of calcium-mediated oxidative stress [63]. In Cav1.3 knockout mice, the IHCs remain immature, because they cannot upregulate voltage- and Ca^2+^-activated K^+^ channels [64]. In zebrafish HCs, the Cav1.3 channels play a role in the regulation of presynaptic size during cell development [65].

### 3.2. Ototoxicity

Some ototoxic drugs can also damage ribbon synapses. High doses of aminoglycosides, including gentamicin and neomycin, induce auditory threshold shifts for HC toxicity [66] by causing acute swelling of SGN terminal dendrites [67, 68]. In the normal cochlea, ribbon synapses accumulate at the base of IHCs and appear in pairs. After aminoglycoside-induced injury, the ribbon synapses move from the base of the IHCs to the perinucleus, and many dissociations of the synaptic pairs are observed [69]. Low-dose gentamicin may cause ribbon synapse-associated protein synthesis disorder (e.g., Ribeye and Otoferlin), which affects the plasticity and neurotransmitter release pattern of the ribbon synapses [35, 70]. IHC ribbon synapses are the primary targets of aminoglycoside ototoxicity, with resultant hearing loss.

Ouabain is also ototoxic. Injecting ouabain through the cochlea round window membrane in mice can selectively destroy type I SGNs. Disintegrated synaptic pairs appear after ouabain injury, and these dissociated synaptic pairs gather in IHCs near the nucleus, which is similar to the characteristic damage of aminoglycosides [71].

Cisplatin can also cause hearing loss. The mechanisms underlying cisplatin ototoxicity include the induction of calcium ion accumulation in IHCs, which prevents calcium influx. The decreasing calcium current leads to dysfunction of synaptic release and impairs vesicle cycling, thereby resulting in hearing loss [72].

### 3.3. Noise Exposure

In humans, intense or prolonged exposure to noise can result in profound hearing loss. However, in some cases, noise exposure only leads to an elevated hearing threshold [73]. There are many related studies on noise-induced sensorineural hearing loss [74–76], because the pathological mechanism of noise-induced hearing loss (NIHL) is extremely complicated, and the exact mechanism is still unclear.

Researchers have shown that severe noise exposure can lead to damage or loss of HCs or HC synapses or permanent threshold shifts [77–79]. Moderate noise exposure results in an elevation in the hearing threshold, but the threshold eventually returns to normal [80]. However, despite the clinical hearing threshold returning to normal, the numbers of ribbon synapses and afferent fiber terminals are significantly reduced [81, 82]. Moderate noise exposure can significantly reduce the number of ribbon synapses per IHC in the apical region, but this number then fully recovers. Tight control of Ca^2+^ influx through VGCCs is important for the precise transmission of auditory signals in IHC ribbon synapses. After moderate exposure to noise, the Ca^2+^ current amplitude decreases but gradually recovers [83]. In IHCs, a temporary decrease in Ca^2+^ current is consistent with a decrease in the number of ribbon synapses. IHCs prolong the exocytosis of synaptic vesicles and are related to the rapid replenishment of these synaptic vesicles [84]. After noise exposure, the fast and efficient recycling of synaptic vesicles decreases, which may contribute to the loss and recovery of ribbon synapses; such a decrease could also be caused by a decrease in the efficiency of Ca^2+^ in triggering synaptic vesicle release. Thus, exploring changes in synaptic function after noise exposure may be a new direction for the study of implicit hearing loss [85].

### 3.4. Aging

Presbycusis, also known as age-related hearing loss, results from age-related decline in the function of the inner ear [86]. Ribbon synapses between IHCs and SGNs are formed as the first afferent neuronal connection in the auditory nervous system. In the early stage of presbycusis, the cochlear ribbon synapse is the primary insult site [87]. A high rate of neurotransmitter release is a characteristic of ribbon synapses. An effective neurotransmitter transport system in IHCs for the maintenance of synaptic vesicle recycling and tonic exocytosis is largely dependent on ATP produced by mitochondria [88]. Increased oxidative damage to mitochondrial DNA and decreased mitochondrial ATP production are detected in the cochlea of mice exposed to D-gal-induced aging. Thus, age-related ribbon synapse insult may result from mitochondrial oxidative damage and subsequent dysfunction [87].

## 4. Synapse Protection

Certain proteins or factors protect ribbon synapses, so targeting these to protect the ribbon synapses would be an effective measure for preventing HHL.

When exposed to noise, glutamate accumulation in the synaptic cleft leads to overactivation of ionotropic glutamate receptors (iGluR) and subsequent excitotoxic damage, which in turn damages the postsynaptic terminal. Thus, regulating glutamate excitotoxicity and blocking iGluR might prevent noise-induced cochlear synaptopathy [89]. The application of the iGluR agonists AMPA, kainic acid (KA), and NMDA to the mammalian inner ear or HC explants can mimic glutamate excitotoxicity associated with noise exposure [90]. In mammals, the application of AMPA or KA leads to overactivation of the iGluR receptors, which affects neurotransmission at postsynaptic afferent terminals [89]. The application of NMDA glutamate receptor blockers, such as kynurenate and DNQX, can reduce the dendritic SGNs. For instance, MK-801, an antagonist for the NMDA subtype of glutamate receptors, is effective against NIHL; however, whether this protection is attributable to the preservation of the synapses between IHCs and SGNs or other mechanisms has yet to be determined [91]. Therefore, detailed pharmacodynamics studies with current immunohistological methods are needed to determine whether modulation of the glutamate excitotoxicity pathways could prevent cochlear synaptopathy.

The distribution of both VGCC blockers (L- and N-type) and calcium channels in IHCs is reportedly consistent. Thus, VGCC blockers could be used to protect the cochlear IHCs from noise damage. However, whether the application of a blocker can prevent noise-induced synaptic damage has yet to be demonstrated. Another protective approach is to regulate calcium signaling pathways. Studies have shown that blockers for T-type VGCCs can protect against NIHL, and L-type VGCC blockers also can be used to prevent NIHL. However, these studies did not focus on cochlear synaptopathy [92, 93].

Recently, many other factors and proteins have been shown to protect synapses. The WW domain-binding protein 2 encoded by WBP2 acts as a transcription coactivator for the estrogen receptor and progesterone receptor [94]. Both estrogen and progesterone can protect against stroke and glutamate toxicity [95]. In the auditory system, estrogen controls central and peripheral auditory processing [96]. According to research, Wbp2 may be a potential target to prevent or even reverse progressive hearing loss via regulating the estrogen signaling pathway, which has vital involvement in synaptic damage [97].

Fibroblast growth factor-22 (FGF22) plays a novel role in protecting the IHC ribbon synapses of the cochlea from gentamycin ototoxicity. In mice, intraperitoneal injection of gentamycin causes a decrease in the ribbon synapse number and FGF22 expression as well as an increase in MEF2D expression. FGF22 infusion reversed the loss of ribbon synapse and upregulation of MEF2D caused by gentamycin and restored hearing [98]. MEF2D, a member of the myocyte enhancer factor 2 (MEF2) family, which is reported to be highly expressed in the brain and is regulated by calcium signaling pathways, suppresses the number of excitatory synapses [99]. Activating FGF22 might provide the conceptual basis for the therapeutic strategies.

Nicotinamide riboside (NR) is a potent stimulator of NAD^+^ production and can increase NAD^+^ levels in many types of animal tissues. NAD^+^ is a fundamental molecule in all mammalian cells and plays an essential role in numerous cellular processes, such as metabolism and cell signaling pathways. The use of NR could promote the recovery of synapses, contributing to the protection of hearing [100].

The macrophage migration inhibitory factor is expressed in the inner ear of mammals or chickens and plays a role in neurite outgrowth and neuronal survival at the stage of initial neurite outgrowth; it is essential for the maintenance of normal hearing [101]. Cyclin-dependent kinase 2 inhibitor kenpaullone may be effective in preventing recessive hearing loss caused by low-decibel noise, although the exact mechanism remains unclear [102, 103].

The network of interactions between these cytokines needs further study, and the molecular mechanisms that regulate inner ear development and innervation also need to be further elucidated.

## 5. Synapse Regeneration

Cochlear ribbon synapses have only limited spontaneous regenerative capacity [104, 105]. Recent studies have shown that many factors and signaling pathways may play a role in promoting synaptic reconstruction and regeneration, which gives new insight into the reversal of hearing loss.

Neurotrophins are critical factors in the mammalian cochlea. Brain-derived neurotrophic factor (BDNF), neurotrophic factor-4/5 (NT-4/5), neurotrophic factor-3 (NT-3), and nerve growth factor play a role in ribbon synapse formation during development, in plasticity, and in the maintenance of synaptic stability [106–108]. NT-3 and BDNF are the two major types [109]. BDNF levels in immature cochleae are higher than those in mature cochleae, in which BDNF expression eventually declines to undetectable levels. Analyses of neurotrophin levels in adult cochleae have detected only NT-3 and glial cell line-derived neurotrophic factor [110–112]. Knockout of BDNF and NT-3 in the inner ear of newborn mammals caused ribbon synaptic damage in the vestibule and inner ear, respectively, resulting in vestibular dysfunction and hearing loss [113]. Application of BDNF and NT-3 promoted reconstruction of SGNs in cultured cochleae, and they expressed markers of postsynaptic ototoxic drug damage [114]. Several studies have reported that synapse regeneration can be promoted by NT-3. Synapse density increased, and the ABR threshold decreased in mice overexpressing NT-3 [112]. NT-3 has a protective ability, as demonstrated in tests of the ABR amplitude and synapse count by round window injection. After noise trauma, applying NT-3 through the round window may be an ideal solution for protecting against synaptic damage [115, 116].

Photobiomodulation (PBM) enhances neural growth and connections in the peripheral nervous system. The use of low-intensity lasers for PBM has been studied in various fields for therapeutic purposes, and some studies indicate that PBM may have therapeutic effects on hearing [109]. PBM promotes and activates cell growth by increasing the mitochondrial membrane potential and ATP production after oxidative stress, which play essential roles in NIHL [110]. A study found that PBM rescued cochlear synaptopathy after acoustic overexposure by increasing neurotrophins and intracellular components, including ATP, matrix metalloproteinases, and Ca^2+^ [111].

Another important synaptotrophic factor is glutamate. Glutamatergic transmission can be mediated by Vglut3 [113]. In Vglut3-deleted mice, the number of newly generated synaptic contacts at the dendrites of SGNs was significantly decreased when compared with the number in normal controls, which indicated that the proper release of glutamate transmitter plays an important role in the regeneration of synaptic contacts *in vitro* [113].

Clarin-1 (CLRN1) is essential for hair bundle morphogenesis in auditory HCs. Usher syndrome type IIIA is caused by mutations in CLRN1 encoding clarin-1 [117]. The protein is crucial for the structural organization and function of the presynaptic Cav1.3 Ca^2+^ channels at the IHC ribbon synapses, as well as for the distribution of postsynaptic AMPA receptors. Loss of CLRN1 leads to disorganization of the synaptic F-actin network in IHCs, which is likely to account for lower Ca^2+^ efficiency in exocytosis in IHCs. Virus-mediated CLRN1 could preserve ribbon synapses and hair bundle structures in CLRN1 mutant HCs, indicating that gene therapy targeting mutant HCs could reverse synaptic defects and restore hearing. Gene therapy approaches could be used to effectively treat genetic inner ear disorders.

## 6. Conclusion

Many researchers have increased awareness of synaptopathy, which may contribute to HHL. Studying synapse formation is especially important for the diagnosis and treatment of HHL. Research progress in the topic of ribbon synapses, as well as analysis of the biological mechanisms in ribbon synapses, is rapid. The mechanisms underlying ribbon synapse damage and protection have been explored in the past decades; however, more studies to identify the survival and regenerative mechanisms of ribbon synapses are needed. Furthermore, implementing large-scale screening of the genes and chemicals involved in ribbon synapses is important in identifying candidate mechanisms that can be manipulated with pharmaceutical, genetic, or other interventions for broader discoveries about ribbon synapses. As biochemical, molecular, and imaging approaches become more advanced, insights into the etiology and mechanisms responsible for the survival, damage, and regeneration of ribbon synapses will be obtained, which may in turn lead to the discovery of novel effective therapeutic targets for HHL.

## Data Availability

No data were used to support this study.
